# Association of MicroRNA-146a with Type 1 and 2 Diabetes and their Related Complications

**DOI:** 10.1155/2023/2587104

**Published:** 2023-03-03

**Authors:** Mahyar Ghaffari, Sara Razi, Hamidreza Zalpoor, Mohsen Nabi-Afjadi, Fariba Mohebichamkhorami, Hakimeh Zali

**Affiliations:** ^1^Department of Biology, Parand Branch, Islamic Azad University, Parand, Iran; ^2^Vira Pioneers of Modern Science (VIPOMS), Tehran, Iran; ^3^Shiraz Neuroscience Research Center, Shiraz University of Medical Sciences, Shiraz, Iran; ^4^Network of Immunity in Infection, Malignancy & Autoimmunity (NIIMA), Universal Scientific Education & Research Network (USERN), Tehran, Iran; ^5^Department of Biochemistry, Faculty of Biological Sciences, Tarbiat Modares University, Tehran, Iran; ^6^Proteomics Research Center, Shahid Beheshti University of Medical Science, Tehram, Iran

## Abstract

Most medical investigations have found a reduced blood level of miR-146a in type 2 diabetes (T2D) patients, suggesting an important role for miR-146a (microRNA-146a) in the etiology of diabetes mellitus (DM) and its consequences. Furthermore, injection of miR-146a mimic has been confirmed to alleviate diabetes mellitus in diabetic animal models. In this line, deregulation of miR-146a expression has been linked to the progression of nephropathy, neuropathy, wound healing, olfactory dysfunction, cardiovascular disorders, and retinopathy in diabetic patients. In this review, besides a comprehensive review of the function of miR-146a in DM, we discussed new findings on type 1 (T1MD) and type 2 (T2DM) diabetes mellitus, highlighting the discrepancies between clinical and preclinical investigations and elucidating the biological pathways regulated through miR-146a in DM-affected tissues.

## 1. Introduction

Diabetes mellitus (DM) is one of the serious persistent concerns confronting healthcare systems across the world [1, 2]. According to the International Diabetes Federation (IDF), this disorder will impact 642 million individuals until 2040 [3]. Diabetes affects 8.8 percent of the adult population globally, pursuant to the IDF. T2DM is the more common type of diabetes; only 10–15 percent of people with diabetes have T1DM [4]. Diabetes is responsible for 6.8 percent of worldwide mortality in the 20–79 age range and is a primary reason for decreased life expectancy [5, 6]. About 10% of diabetic patients have type 1 diabetes (T1D), which is characterized by an uncontrolled and destructive immune response to pancreatic beta cells [7, 8], whereas the remaining DM patients have type 2 diabetes (T2D), whose main risk factors include overweight, insufficient physical activity, and smoking [9, 10]. Numerous clinical studies have shown that lifestyle modifications may successfully prevent the progression of T2D. Genetic variables, on the other hand, have been found to be key regulators of individual vulnerability to T2D development and responsiveness to lifestyle treatments [11]. These findings suggest the need for further study into the molecular roots of diabetes and the identification of additional variables implicated in the disease's etiology. In this regard, microRNAs (miRNAs) have received a lot of attention recently as unique fine modulators of gene expression [12]. miRNAs attach to the mRNA 3'UTR and inhibit translation or assist mRNA cleavage, suppressing gene expression [13].

Several mRNAs of mammals have been shown to be suppressed by miRNAs, and dozens of such bindings have been identified so far. A large body of evidence suggests that miRNAs play critical roles in human development and illness by fine-tuning gene expression [14]. The importance of miRNAs in regulating immune cell differentiation and function is underscored by the phenotypic disturbances that occur when miRNA expression is altered. Considering the role of miRNAs in modulating immune responses, it is likely that any disruption in the expression of miRNAs contributes to the pathogenesis of autoimmune diseases, chronic inflammation, and malignancies. Currently, dysregulated miRNA expression is associated with several human diseases [15, 16]. MiR-146a has recently been shown to be an important modulator of innate as well as adaptive immune cell differentiation and function. miR146a participates in the regulation of multiple physiological responses by targeting specific messenger RNAs to repress their translation. Also, by acting as a negative regulator of transcription, it plays an important role in inhibiting the escalation of inflammatory responses and maintaining immune homeostasis [17]. In summary, in this review, we aimed to review the potential pathological functions of miR-146a in the pathogenesis of DM and its related complications.

## 2. Biogenesis of miR-146a

MicroRNA 146a, which is encoded by the MIR146A gene located on chromosome 5q33.3, is a small noncoding RNA [18].

**Figure 1:**
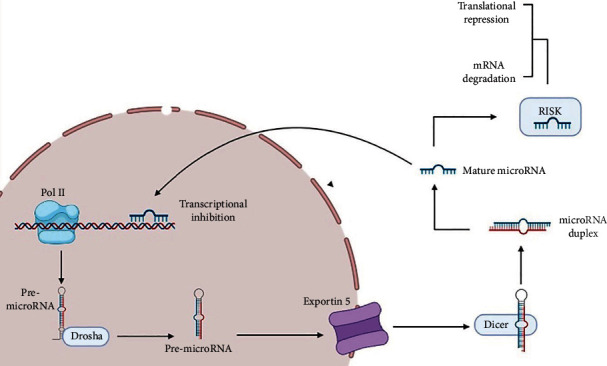
The process of microRNA transcription, maturation, and function in the cell.

Like the maturation process of other miRNAs, miR-146 has the same standard procedure of miRNA biosynthesis pathways (Figure 1), which begins with transcription of pri-miRNA and is accompanied through its cleavage with the microprocessor complex in the nucleus, including the DGCR8 and Drosha proteins, that results in the generation of a precursor miRNA with 60–70 nucleotides and stem-loop form as pre-miR-146a [19]. Pre-miR-146a molecules are cleaved once more by Dicer after being exported by exportin-5 from the nucleus into the cytosol, leading to the generation of RNA ~22 nucleotide duplexes [20]. TAR-RNA binding protein (TRBP), also termed TARBP2, and then interacts with DICER1. DICER1 is not involved in pre-miRNA maturation, whereas TRBP enhances the precision of DICER1-mediated cleavage of pre-miRNAs in a structure-dependent way and alters the identification of miRNA guide strands by forming iso-miRNAs that are one nucleotide longer than regular miRNAs [21]. Argonaute protein interacts with short duplexes of RNA, creating the backbone of a multi-sub-assemblies complex known as the RNA-induced silencing complex (RISC) and producing single-stranded RNAs, having the ability of mRNA interaction [22]. Interleukin-1 receptor-related kinase 1, tumor necrosis factor transcript, interleukin-1-beta, TNF receptor-related factor 6, and complement factor H are some of the targets of miR-146a [23] (Figure 1).

### 2.1. miR-146a and Type 1 Diabetes Mellitus (T1DM)

T1DM results from uncontrolled and extended immune responses of immune cells on *β*-cells, and resultant diabetes progresses due to the gradual loss of insulin-producing cells [24]. MicroRNA 146a is a small noncoding RNA that in humans is encoded by the MIR146A gene, which is located on Chr 5: 160.49–160.49 Mb. MicroRNAs are short noncoding RNAs that are involved in posttranscriptional regulation of gene expression in multicellular organisms by affecting both the stability and translation of mRNAs [25]. miR-146a is a key regulator of inflammatory processes. Expression of miR-146a is altered in target organs of diabetic complications, and deficiency of miR-146a has been implicated in their pathogenesis [26]. In human monocytes, miR-146a, which is encoded by the locus of the LOC285628 gene located on chromosome 5, reacts to lipopolysaccharide (LPS) stimulation, and its activation is reliant on nuclear factor kappa B (NF-*κ*B) [27]. The presumptive function of miR-146a is based on this pathway, in which its upregulation via NF-*κ*B signaling provokes it to direct downregulation of its target genes such as TNF receptor-associated factor 6 (TRAF6) and IL-1 receptor-associated kinase 1 (IRAK1), leading to the dampening or termination of an excess inflammatory response through a negative control feedback loop [28]. The NF-*κ*B response to LPS in monocytes and DCs is a phenotypic and probable pathogenic marker for human T1DM [29]. In T1D animal models, TRAF6 mediated high glucose-induced endothelium damage through NF-*κ*B and AP-1-dependent signaling [30].

Because T1D is frequently associated with other autoimmune diseases (AID), some studies have also been published on the role of epigenetics in autoimmune diseases and T1D extrapancreatic autoimmune disease. For instance, Milluzzo et al. investigated 115 T1D patients that were sporadic cases (S-T1D) and 115 familial cases (F-T1D). The results indicated that the F-T1D group had a higher percentage of AIDs, a significant earlier onset of AIDs at Cox regression analysis, and the highest prevalence of both additional organ-specific antibodies and overt AIDs than the S-T1D group [31]. These observations could be due to the miRNAs as crucial modulators of immune cell functions, thus representing major players in the regulation of immune homeostasis. In particular, miRNAs are associated with many facets of immune responses such as development, activation, and differentiation [32, 33]. To prove this statement, some studies deleted the factor genes associating in miRNAs maturation in immune cells. For example, in a study, Dicer, Drosha, or Argonaute deletion in T cells lead to block interferon-*γ* (IFN-*γ*) production [32, 33].

It is also worth noting that miR-146a expression has been linked to elevated levels of glutamic acid decarboxylase antibodies (GADA) and is lowered in peripheral blood mononuclear cells (PBMC) of individuals affected with T1DM. Furthermore, lncRNA SRA was shown to have a role in T1D pathogenesis by inhibiting miR-146b in *β*-cells and increasing the IRAK1/LDHA/pLDHA signaling pathway, terminating in increased reactive oxygen species (ROS) generation [34]. Liu et al. established that miR-146a may operate as a possible circulating biomarker by demonstrating a decreased expression pattern in the blood of T1DM patients in comparison with normal controls in bioinformatics and clinical analysis [35].  Table 1 summarizes multiple clinical studies about the association of miR-146a with T1D.

The correlation between miR-146a and T1D has recently ended in research concerning its polymorphisms. In this case, case-control research found that the C/G allele for the polymorphism of rs2910164 in miR-146a was linked with protection against T1D [36]. The rs2910164 (G/C) polymorphism is found in the seed sequence of pre-miR-146a [37]. Because miR-146a was reduced, its target genes, such as IRAK-1 and TRAF-6, were less effectively inhibited. This polymorphism, however, was not linked to diabetic retinopathy in T1DM patients [36]. Barutta et al. also discovered a negative and independent correlation between miR-146a-5p and diabetic-related chronic complications, including cardiovascular disorders and diabetic retinopathy, indicating that miR-146a-5p can be potentially considered a biological marker in T1DM complications [26].

### 2.2. miR-146a and T2DM

In patients with T2D, deregulation of miR-146a has been frequently reported, however, there is conflicting information about the direction of deregulation events (up or down). In this section, we focus on investigating the functional role of miR-146a in T2D, innate immunity, the inflammatory response, viral infection, and human diseases. These studies on the expression pattern of miR-146a in T2DM individuals yielded contradictory findings. Balasubramanyam et al. discovered that individuals with T2D had a two-fold drop in miR-146a levels, leading to dramatically elevated TRAF-6 mRNA expression. The presence of the elevated miR-146a expression in the PBMC of T2D patients suggests that the circulating expression of miR-146a can be considered as a prognostic and diagnostic marker in these individuals [39]. Baldeo'n et al. also found that miR-146a expression in blood considerably decreased in patients with T2D compared to nondiabetic controls in a study of Ecuadorian T2D cohorts. They also discovered that this drop was related to augmented amount of proinflammatory blood cytokines including HGF and IL-8 (a vascular/insular repair factor) as distinguishing indicators of glucose control dysfunction. Alipoor et al. in a meta-analysis study found the same reduced level of miR-146a in PBMC and plasma of patients with T2D; however, it was not seen in the patients' serum or plasma samples [40]. García-Jacobo et al. verified the reduced expression of miR-146a. They also addressed miR-34a's connection to miR-146a and resistance to insulin. Furthermore, they stated that the expression of miR-146a in individuals susceptible to diabetes, patients with T2D tolerating insulin therapy, high obesity/glycemia, and T2D patients with diabetic foot ulcers and nephropathy was reduced in serum but was not linked with cell functionality [41]. Habibi et al. also found a substantial drop in miR-146a and a rise in IRAK1, NF-*κ*B, and TRAF6 gene mRNAs in the hippocampus part of rats with diabetes, along with enhanced activity of NF-*κ*B and apoptosis levels in this group in comparison with the control group [42]. In addition, Yavari et al. found that troxerutin pretreatment of rats lowered the levels of mRNAs for IRAK-1, NF-*κ*B, and TRAF-6 in the hippocampus of both diabetic and nondiabetic animals, potentially because of a negative control feedback loop mediated by miR-146a [43].

The expression level of miR-146a is variable in response to physical/medical treatments, age, sex, or the presence of a specific polymorphism. In this example, older T2D patients were given acute strength and cardiovascular exercise, and the amount of miR-146a expression in their blood was examined. As a result, diabetic patients had a greater drop in serum blood glucose than nondiabetics, with a significant reduction following the strength training intervention, which was accompanied by a favorable change in the whole blood circulation levels of miR-146a but not the other miRNAs [44, 45]. In this respect, it is possible to infer that miR-146a levels in peripheral blood leukocytes are inversely related to insulin resistance. In an in vivo study, Alipour et al. discovered a substantial rise in the expression levels of miR-146, NF-*κ*B, and inflammatory cytokines (TNF-*α*, IL-6, and IL-1), while a significant reduction in the pancreatic expression levels of TRAF6 and IRAK1 was found in the diabetes group in comparison with the control group. Swimming exercise, on the other hand, led to a substantial drop in the expression levels of miR-146a, NF-*κ*B, and inflammatory cytokines and a significant rise in the expression levels of TRAF6 and IRAK1 in the exercise-diabetes group as compared to the diabetic group [46]. On the other hand, microRNA (miRNA) polymorphisms, as a new mechanism, are linked to pathological conditions through interference with miRNA activities [47]. Investigating the rs2910164 polymorphism in miR-146a generated contradictory results. In this situation, Ciccacci et al. and Wang et al. in independent cohort studies on Italian and Chinese T2D patients, respectively, reported that miR-146a had no significant link with illness initiation and that polymorphism in the-miR-146a had no significant differences between cases and controls [47, 48]. Alipoor et al., on the other hand, revealed that the polymorphism of rs2910164 in miR-146a may be related to T2D and its cardiovascular risk factors in an Iranian population [49]. Additionally, Shankaran et al. demonstrated in a meta-analysis that the existence of the C allele in the miR-146ars2910164 polymorphism may have a function as a risk factor in the Indian population of T2D patients via modifying miR-146a maturation [50]. Eventually, a complete meta-analysis of the aforementioned studies revealed that the polymorphism rs2910164 is not associated with T2D susceptibility [51]. In terms of the rs2910164 polymorphism (G/C), it was discovered that the rs2910164-C allele is related to lower levels of expression of miR-146a [52]. Mirzaie et al. found that levels of miR-146a were considerably decreased in diabetes affected patients than in healthy controls that are negatively associated with levels of HbA1c and fasting blood glucose in males when they looked at the association of age and sex with miR-146a expression in T2D patients [53]. Furthermore, in women, miR-146a circulating levels correlated negatively with uric acid, azotemia, ferritin, and waist/hip ratio. Circulating miR-146a might be employed being age-related, potential biomarker in the aging rout of healthy/unhealthy individuals in this scenario [54]. LncRNAs may also negatively or positively affect the development of certain diseases. Chen et al. discovered that LncRNA PTGS2 overexpression is observable in T2D patients. They also discovered that this overexpression may impair islet-cell function and that knocking it down can result in an increase in miR-146a-5p expression. The miR-146a-5p overexpression may limit RBP4 expression through a cascade route. The elevated RBP4 plasma levels were linked to diabetic retinopathy and vision-threatening diabetic retinopathy in Chinese T2D patients, suggesting a role for RBP4 in the pathophysiology of diabetic retinopathy problems [55]. Concerning contradicting findings, Shokri-Mashhadi et al. reported the enhanced level of miR-146a in plasma levels of T2D patients and that astaxanthin supplementation might lower plasma levels of this miRNA [56]. On the other hand, Zeinali et al. discovered that mir-146a levels were decreased in T2DM patients and prediabetic individuals related to healthy controls by assessing the plasma expression level of mir-146ain diabetic patients and prediabetic individuals [57].

Endothelial-derived miR-146a regulates the increased production of ECM proteins and inflammatory cytokines in the retina and kidneys in diabetes [58, 59]. According to Qin et al. study, the miR-146a level in exosome of bone marrow derived mesenchymal stem cells can restore *β*-cell function in T2D rats through the NUMB/*β*-catenin signaling pathway. All of these suggest that miR-146a overexpression might be a unique method for treating T2D [60]. The usage of bone marrow mesenchymal stem cells (BM-MSCs)-derived exosomes containing miR-146a has also been shown to contribute to the healing of the hippocampus damage induced by diabetes and, as a result, to the recovery of cognitive impairment [61].

### 2.3. miR-146a and Diabetic Patients with COVID-19

Nowadays, the pandemic of coronavirus disease-2019 (COVID-19) is a serious hazard for the healthcare system and patients with primary diseases [62–68]. It has been revealed that severe acute respiratory syndrome coronavirus 2 (SARS-CoV-2) infections has the ability to lead to an increased host immune system response and severity of multiple diseases, such as diabetes, hypertension, cardiovascular, and neurological complications [69–73]. During human coronavirus infection, miRNAs have a critical role in regulating the host's innate immune system [70].

It is intriguing that the relationship between diabetes, miR-146a, and COVID-19 may be a hot point. In this regard, it is indicated that individuals with diabetes, overweight, and high blood pressure have lower levels of circulating miR-146a, which may be aggravated by SARS-CoV-2 infection since the virus may interact with miR-146. Downregulation of miR-146a might be one of the mechanisms generating severe COVID-19 because of an insufficient host antiviral response, characterized by more protracted and inappropriate cytokine production and an absence of a feedback mechanism to control inflammatory damage to tissues. As a result, miR-146 reduction in diabetes can cause excessive inflammation (due to inadequate inhibition of IRAK1/TRAF6), increased fibrosis (due to fibronectin overexpression), and increased MCP-1 production, followed by further reduction of miR-146a (due to enhanced TGF-*β*1/ErbB4/Notch1 signaling), which can eventually terminate in severe COVID-19 [70]. To further elucidate its molecular pathways, it should be noted that factors responsible for entrance of SARS-CoV-2 in to cells are expressed at varying levels in masticatory mucosa (tongue) and salivary glands, which are equivalent to those observed in the bronchi and tonsil [74].

Consequently, diabetes-induced increasing in miR-146a, which is plays and important role in cell entrance regulation of SARS-CoV-2 elements and overexpression of angiotensin-converting enzyme 2 (ACE2). It regulates genes associated with host immune response, is expected to upregulate angiotensin-converting enzyme 2 expression, a crucial receptor for the entrance of SARS-CoV-2, and modulate the response of host against viruses.

## 3. Pathological Roles of miR-146a in Diabetes Mellitus Complications

**Figure 2:**
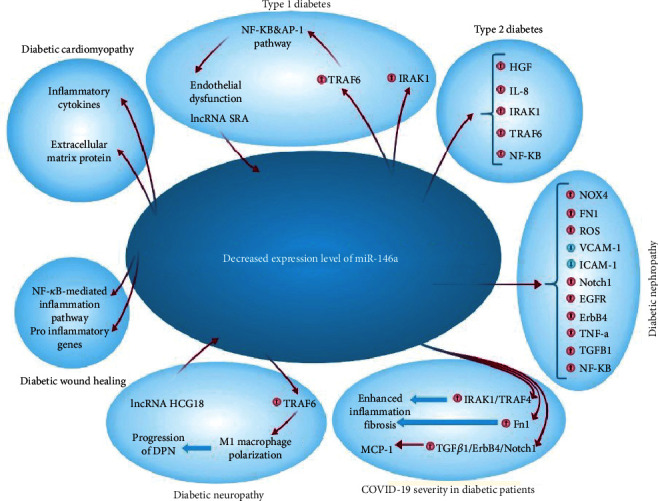
The decreased expression levels of miR-146a and its role in diabetes progression.

Various investigations have shown that miR-146a possess a significant role in progression of diabetes complications including nephropathy, wound healing, diabetic olfactory dysfunction, neuropathy, and retinopathy (Figure 2).

### 3.1. Diabetic Nephropathy Pathogenesis and miR-146a

**Figure 3:**
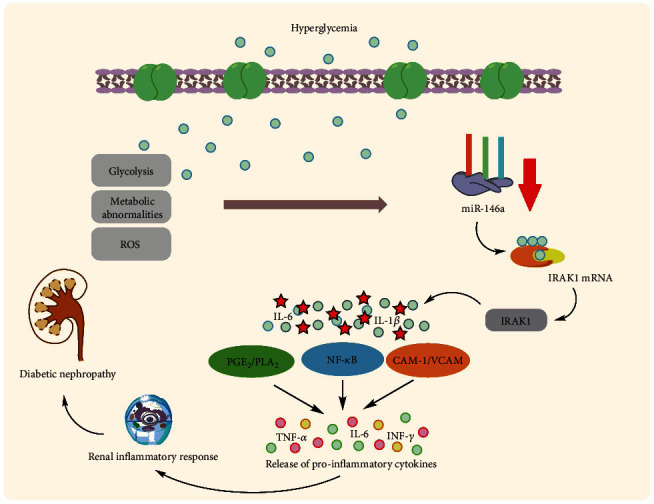
Association of miR-146a with diabetic nephropathy.

Uncontrolled hyperglycemia is a key cause of renal dysfunction in diabetes, activating the glomerular endothelium and causing podocyte activity failure [75]. The inflammatory reaction leads to the activation and proliferation of mesangial cells of the renal glomeruli, which causes the excess generation of ECM. This results in the contraction of podocytes and capillaries and then, decreases filtration rate of glomeruli [76]. Glomerulosclerosis consequently causes destruction of epithelium in tubules with permanent fibrotic alterations [77]. According to Feng et al., an animal investigation found that miR-146a was lowered in the renal tissue of type 1 and 2 diabetic rats, resulting in increased expression of fibronectin, an essential component of the extracellular matrix. However, they demonstrated that intravenous injection of miR-146a mimics in type 1 and 2 diabetic rats may reinstate retinal miR-146a expression and reduce fibronectin rate in diabetes but not kidneys, and additional investigations are needed to establish this therapeutic method in diabetic nephropathy [58, 78]. Wang et al. identified nicotinamide adenine dinucleotide phosphate (NAPDH) oxidase 4 as a new target for miR-146a (NOX4). They discovered that decreased expression of miR-146a and consequent upregulation of NOX4 expression can result in an augmented rate of ROS production, oxidative stress, and inflammation, as well as suppressed expression of intracellular adhesion molecule-1 (ICAM-1) and vascular cell adhesion molecule-1 (VCAM-1) proteins, leading to diabetic nephropathy pathogenesis in diabetic rats [79]. Lee et al. validated this decreased expression in diabetic kidneys in the glomeruli podocytes of T2D humans and diabetic mice, which correlates with higher albuminuria and glomerular damage [80]. This decreased expression has been linked to downstream targets of miR-146a, including Notch-1, ErbB4, and EFGR. This downregulated expression pattern was found to be up to five times more expressed in diabetic nephropathy patients' kidney samples as compared to controls. In vitro investigations on human renal glomerular endothelial cells revealed that hyperglycemia elevated TGF-*β*1, TNF-*α*, and NF-*κ*B expression [81] (Figure 3). Not only do renal endothelial cells and podocytes have lower expression of miR-146a in diabetic nephropathy, but other immune cells may also have this expression profile and potentially harm the kidney by creating a proinflammatory microenvironment. Keeping this in mind, Bhatt et al. discovered that in diabetes animal models, miR-146a expression was enhanced in both peritoneal and intrarenal macrophages. Remarkably, miR-146a reduction throughout diabetes resulted in enhanced expression of M1 activation markers and repression of M2 markers in macrophages [82]. In contrast to the above studies, Alipour et al. revealed contradictory findings in which miR-146a was elevated in diabetic kidneys of rats compared to normal controls [83]. The C allele of rs2910164 in miR-146a was reported to be related to microvascular diabetic nephropathy problems in T1D patients in Caucasian individuals. The results show that this is mostly due to a deficiency in the pre-maturation level of miR-146a [84].

### 3.2. miR-146a and Pathogenesis of Diabetic Neuropathy

**Figure 4:**
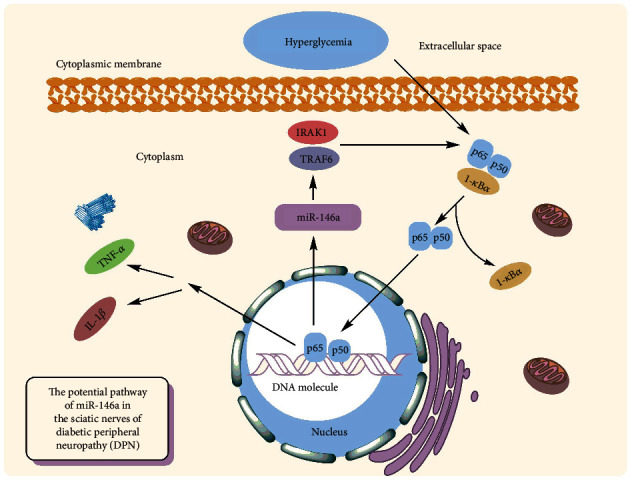
Association of miR-146a with diabetic neuropathy.

Diabetic peripheral neuropathy (DPN) is one of the most common complications of T2DM [85]. The reported prevalence of DPN ranges from less than 5% to 60%, with an average of 26.4 percent [86]. The inflammatory responses have been linked to the evolution of diabetic nephropathy, with significant levels of inflammatory cytokines seen in peripheral nerve tissues in diabetic nephropathy models of rats [87]. Reduced production of an anti-inflammatory miRNA, such as miR-146a, might be a critical piece of this complex puzzle. It has been demonstrated that the hyperglycemia leads to reduced expression of miR-146a while it causes increasing levels of TRAF6 and IRAK1 in neurons of dorsal root ganglion (DRG) [88] (Figure 4.). In vitro studies have been demonstrated that miR-146a mimics may significantly inhibit hyperglycemia-induced neuronal death [89]. The same findings were obtained from diabetic rats' sciatic nerves, confirming its protective action against diabetic neuropathy and DPN. Wang et al. discovered that a small polypeptide with acidic phase called thymosin-4, which has numerous roles such as neurorestoration and anti-inflammation, may raise miR-146a levels and overcome the impact of diabetes on DRG neurons in diabetic mice. This beneficial effect was attributed to neurovascular reorganization and the suppression of proinflammatory signals [90]. Similar findings were obtained by Luo et al. using the nanoparticle–miRNA-146a-5p polyplexes in addition to thymosin-4. In this scenario, nano-miR-146a-5p was proven to increase nerve conduction velocity while also alleviating demyelination and morphological injuries in the sciatic nerve of the DPN rats. The protective effects of nano-miR-146a-5p against neural cell death were attributable to reduced production of proinflammatory cytokines and caspase-3 in sciatic nerve cells, as well as enhanced expression of myelin basic protein. Furthermore, systemic injection of miR-146a mimics to diabetic mice was demonstrated to slow the course of DPN, resulting in increased intraepidermal nerve fibers, myelin thickness, and axonal diameters of sciatic nerves [91]. In silico investigation revealed that lncRNAs XR 598132, XR 351905, XR 357013, XR 589615, XR 589933, XR 600244, XR 353891, and XR 595664 control miR-146a in sciatic nerve cells. It is hypothesized that dysregulation of the aforementioned lncRNAs may contribute to the etiology of DPN [92]. In addition, dysregulation of neuronal regulatory lncRNAs miR-146a in may halt the progression of diabetic neuropathy; it can accelerate the disease too. According to it, lncRNA HCG18 has been demonstrated to increase M1 macrophage polarization by targeting miR-146a and upregulating TRAF6 expression, hence enabling DPN development [85]. The overexpression of miR-146a seems to slow the course of diabetic neuropathy, and exosomes have been proposed as potential therapeutically means for delivering miRNAs. Fan et al. discovered that MSC-exosomes containing miR-146a may reduce peripheral blood inflammatory monocytes and endothelial cell activation through inhibiting the signaling pathway of Toll-like receptor (TLR)-4/NF-*κ*B [93].

### 3.3. miR-146a and Wound Healing

Diabetic wound healing has been defined by diminished chemokine production, poor angiogenesis, and an aberrant inflammatory response [94, 95]. A growing amount of available data shows that continuous elevation of inflammatory gene expression may lead to the pathophysiology of chronic diabetic wounds by activating inflammatory pathways. Delayed wound healing can also affect the diabetic eye (cornea), resulting in vision impairment [96]. Unlike prior findings, Winkler et al. demonstrated that miR-146a has enhanced in diabetes limbus compared to the normal limbus, resulting in delayed wound healing of epithelial cells in limbal in vitro and faster wound healing in cultured human diabetic derived corneas [97]. Unlike previous research, Xinling et al. discovered that skin wound healing was delayed in miR-146a knocked-out mice by boosting inflammatory responses, indicating that it can be targeted to expedite wound healing [98]. Additionally, it has been found that miR-146a is dramatically downregulated in diabetic mouse wounds. This downregulation was found to be closely associated with the enhanced level of gene expression of its proinflammatory target genes. Employing the mesenchymal stem cell (MSC) therapy, Xu et al. discovered that MSC medication was related to a sharp rise in expression of miR-146a and reduced expression level of its proinflammatory target genes [99]. Nanotechnology-based treatment appears to be promising because nanoparticles are among the most effective biological vehicles for delivering noncoding RNAs. Dewberry et al. also found that coupling miR-146a to cerium oxide nanoparticles effectively improved wound healing [100]. The team of Lindel et al. also confirmed the previous study and mentioned that intradermal injection of conjugated miR-146a to cerium oxide nanoparticles increases wound collagen production, improves angiogenesis, and reduces inflammation and oxidative stress, promoting faster wound repair in diabetic wounds [101]. Curcumin is an excellent wound healing medication [102]. Huang et al. discovered that a curcuminoid derivative can expedite diabetic mice's skin wound healing by boosting miR-146a and blocking the NF-*κ*B-mediated inflammatory pathway [103].

### 3.4. miR-146a and Diabetic Retinopathy

Diabetic retinopathy (DR) affects roughly 80% of people with T1D or T2D who have been diagnosed for at least 20 years [104]. In several studies, miRNAs have been shown to play different roles in developing DR and have also shown an association between some miRNAs with the grade of DR [105–107]. DR begins with lesions that are nonproliferative such as changed permeability of vessels and blood flow in retina, and thickening of basement membrane, depletion of pericytes, and the creation of capillaries with no cells, producing macular edema. Later, the illness progresses into a pathological proliferative and neovascularization stage: the arteries expand into the vitreous evoking retinal detachment and hemorrhage which leads to vision loss [108]. For the first time, Feng et al. verified that miR-146a was concentrated in endothelial cells of the retina and was lowered in diabetes. Intravenous injection of miR-146amimics recovered miR-146a in retina and lowered fibronectin synthesis in diabetes [78]. Besides that, Wang et al. stated that diabetes-induced deregulation of miR-146a daily rhythms and inflammatory pathways under miR-146a control has potential implications for the development of DR, implying that miR-146a as a biomarker could be a novel therapeutically target in the DN treatment [109]. Gong et al. discovered a new target of miR-146ain DR pathogenesis from a molecular mechanism standpoint. Roundabout 4 (ROBO4), a major component of angiogenesis, and hypoxia-inducible factor-1 alpha (HIF-1*α*) are targets for miR-146a, and decreased miR-146a may result in decreased cell survival, augmented permeability, and enhanced cell motility [110]. Rasoulinejad et al. also introduced Nrf2, which may contribute to higher inflammation and oxidative stress rate in diabetic retinopathy [111]. Elevated extracellular matrix protein synthesis (such as collagen IIV and fibronectin) in diabetic mice retina was another involvement of miR-146a in the etiology of diabetic retinopathy. This is because retinal tissue dysfunction was minimized in miR-146a overexpressing transgenic mice [58]. Barutta et al. also revealed that blood levels of miR-146a-5p were lowered in diabetic retinopathy patients, which may be used as a new biological marker in the early identification of these individuals [26].

### 3.5. Association of miR-146a and Other Diabetic-Related Complications

Olfaction plays an important function in an individual's diet and social behaviors. The olfactory bulb (OB) is an essential brain lube for odor sensing in which sensory neurons of the olfactory epithelium connect with mitral cells and convey information to other areas for odor interpretation such as the piriform cortex, amygdala, and entorhinal cortex [112]. T2DM patients also have olfactory changes, such as an increased odor perception threshold, decreased odor identification, and a proclivity to develop anosmia. Jiménez et al. discovered that olfactory impairment in T2DM rats is related with IL-1-mediated inflammation and miR-146a overexpression, implying that high levels of IL-1 might cause miR-146a upregulation as negative feedback to inflammatory response in the T2DM rats' olfactory lobe [113].

## 4. Conclusion and Future Directions

One of the most significant long-term challenges facing global healthcare systems is DM. In addition, during the COVID-19 pandemic, diabetic patients are more prone to experience severe symptoms of COVID-19. During multiple studies, microRNAs have been identified as key regulators of DM and related complications [105, 106, 114]. Biological samples and laboratory procedures need to be standardized, and the obtained data need to be confirmed in independent cohorts. For instance, in clinical practice, instruments (fundus oculi, fluorangiography, and optical coherence tomography) are used to detect DR early, even though the timing and modalities of such screening procedures are often not applied properly. However, teleretinal evaluations could improve patients' compliance with screening programs, particularly during the COVID-19 pandemic [105]. It is undeniable that integrating different epigenetic biomarkers, potentially correlated with clinical, instrumental, and biochemical features, will be helpful to identify accurate panels for DR and other DM-related complications prediction. In the future, further prospective studies are required to achieve this aim. Based on the evidence, miR-146a can reduce the progression of diabetes complications, such as nephropathy, retinopathy, neuropathy, olfactory dysfunction, cardiovascular disorders, and wound healing. In diabetic patients, the miR-146a levels are low, which may be aggravated by SARS-CoV-2 infection, since the virus may interact with miR-146a. It is possible to use miR-146a as a marker to predict diabetes clinical outcomes. Therefore, a better understanding of the multiple pathological mechanisms that inhibit miR-146a expression could open new windows in the treatment of diabetes and related complications. Furthermore, we suggest that combinational therapy of miR-146a with conventional and unconventional antidiabetic drugs potentially can be beneficial to combat T1D and T2D and related complications. In this line, Zgheib et al. have found that miR-146a conjugated with cerium oxide nanoparticles (CNP-miR146a) can play a significant role in the treatment of diabetic wounds [100]. Thus future investigations are needed to evaluate the effects of miR-146a combinational therapies.

## Figures and Tables

**Table 1:** Clinical investigations on the correlation of miR-146a with T1D.

Type of diabetes	Tissue	Main findings	Ref.
T1D patients and normal controls	PBMC	Downregulation of miR-146a and overexpression of GADA	[38]
T1D patients and healthy controls	Plasma and PBMC	Increased expression of lncRNA SRAs may downregulate miR-146ain *β*-cells of T1D individuals	[34]
T1D, T2D patients and nondiabetic controls	Serum	Decreased miR-146aexpression in the serum of patients may serve as a potential biomarker via bioinformatic tools accompanied by clinical analysis	[35]
T1D patients and nondiabetic controls	Serum	C/G allele in the rs2910164 polymorphism is correlated with protection against T1D's	[36]

T1D: type 1 diabetes; GADA: glutamic acid decarboxylase antibody; PBMC: peripheral blood mononuclear cells.

## Abbreviations

DM: Diabetes mellitus
T1MD: Type 1 diabetes mellitus
T2DM: Type 2 diabetes mellitus
miRNA: MicroRNA
lncRNAs: Long noncoding RNAs
TRBP: TAR-RNA binding protein
RISC: RNA-induced silencing complex
NF-*κ*B: Nuclear factor kappa B
TRAF6: TNF receptor-associated factor 6
IRAK1: IL-1 receptor-associated kinase 1.
